# Oncological Safety of High Hydrostatic Pressure Treatment: Effects on Cancer-Associated Fibroblast-like Transdifferentiation of Adipose Stromal Cells

**DOI:** 10.3390/cimb48010091

**Published:** 2026-01-16

**Authors:** Julia Kristin Brach, Vivica Freiin Grote, Anika Jonitz-Heincke, Rainer Bader, Daniel Strüder, Marco Hoffmann, Sven Gerlach, Petra Fischer, Markus Wirth, Tim Ruhl, Justus P. Beier, Agmal Scherzad, Stephan Hackenberg

**Affiliations:** 1 Department of Otorhinolaryngology, Phoniatrics and Pediatric Audiology, RWTH Aachen University Hospital, 52074 Aachen, Germany; 2 Center for Integrated Oncology (CIO), RWTH Aachen University Hospital, 52074 Aachen, Germany; 3 Research Laboratory for Biomechanics and Implant Technology, Department of Orthopedics, Rostock University Medical Centre, 18057 Rostock, Germany; 4 Department of Otorhinolaryngology, Head and Neck Surgery “Otto Körner”, Rostock University Medical Center, 18057 Rostock, Germany; 5 Department of Urology and Pediatric Urology, RWTH Aachen University Hospital, 52074 Aachen, Germany; 6 Department of Plastic Surgery, Hand Surgery–Burn Center, RWTH Aachen University Hospital, 52074 Aachen, Germany; 7 Department of Oto-Rhino-Laryngology, Plastic, Aesthetic and Reconstructive Head and Neck Surgery, University of Wuerzburg, 97080 Wuerzburg, Germany

**Keywords:** high hydrostatic pressure, head and neck cancer, squamous cell carcinoma, adipose stromal cells, cancer associated fibroblasts, tumor secretome, cytokine effects

## Abstract

Oncological safety is essential for autologous reconstruction after resection of cartilage-infiltrating head and neck tumors. High hydrostatic pressure (HHP) enables complete devitalization of tumor-infiltrated tissue while preserving extracellular matrix integrity. However, residual soluble tumor-derived products may influence infiltrating stromal cells. This study examined whether conditioned media (CM) from HHP-treated head and neck squamous cell carcinoma (HNSCC) cells induce cancer-associated fibroblast (CAF)-like transdifferentiation of human adipose stromal cells (hASCs). HASCs were exposed to CM from untreated or HHP-treated (300 MPa) HNSCC cells, tumor-CM (TCM), or TGF-β1. Morphological changes in hASCs were evaluated, and CAF marker expression was analyzed by qRT-PCR, immunofluorescence, Western blot, and ELISA. Cytokines were quantified via multiplex analysis. TGF-β1 induced a CAF-like phenotype with α-SMA upregulation, whereas TCM and 0 MPa-CM caused only modest increases in selected markers. Although 300 MPa-CM did not induce CAF-associated molecular signatures, hASCs exhibited morphological alterations, underscoring that morphology alone is insufficient to define CAF transdifferentiation. Cytokine secretion was elevated in response to all CM conditions. These findings indicate that HHP treatment at 300 MPa abolishes the paracrine CAF-inducing potential of tumor-derived mediators in vitro, supporting the oncological safety of HHP-treated tissues under these experimental condition, although further in vivo validation is warranted

## 1. Introduction

High hydrostatic pressure (HHP) treatment is an emerging technique for tissue devitalization that applies pressures of up to 600 MPa to disrupt cellular membranes while preserving the extracellular matrix and the mechanical properties of the graft [1,2,3,4,5,6,7]. Its uniform pressure distribution according to Pascal’s law enables standardized processing of complex tissues, including tumor-infiltrated cartilage [2,5,8]. Beyond safe devitalization, HHP-treated tumor cells show an immunogenic cell death and retain antigenicity, making this approach suitable not only for reconstructive purposes but also for potential cancer vaccination strategies [9,10,11]. Previous work from our group demonstrated that HHP ≥ 300 MPa for 10 min effectively devitalizes head and neck squamous cell carcinoma (HNSCC) cells while preserving the structural integrity of cartilage grafts [11,12]. 

The composition of soluble factors is an important determinant of the biological response to HHP-treated tissue, as these mediators may critically influence surrounding stromal and residual tumor cells. In earlier studies, we observed that HHP treatment of HNSCC cells causes the release of intracellular cytokines into the extracellular milieu [13]. Specifically, interleukin (IL)-1α and IL-1β are released in a pressure-dependent manner and remain present at high concentrations after treatment [13]. Additionally, IL-6 and IL-8 have been detected in the conditioned medium (CM) of HHP-treated HNSCC cells but at lower levels than in untreated controls [13]. Functional assays have revealed that the cytokines present in the supernatants promote proliferation, migration, and invasion of viable tumor cells [13]. However, a direct link between cytokine release and pressure treatment has not been established. As a further step in evaluating the oncological safety of HHP-treated tissues for autologous reimplantation, it is crucial to determine whether residual cytokines in the post-treatment tumor secretome affect non-malignant cells in the tumor microenvironment (TME). 

Within the TME, stromal cells, particularly cancer-associated fibroblasts (CAFs), play a pivotal role in tumor progression. CAFs enhance invasion, angiogenesis, and immune evasion through extracellular matrix remodeling and secretion of tumor-promoting cytokines and chemokines, epithelial-to-mesenchymal transition (EMT), and therapy resistance, ultimately fostering a permissive microenvironment for tumor growth [14,15,16]. Moreover, several studies have demonstrated that a high abundance of CAFs, often reflected by increased stromal myofibroblast density, is associated not only with poor overall survival but also with a higher likelihood of disease recurrence [17,18]. Importantly, CAFs represent a highly heterogeneous cell population, encompassing multiple functional subtypes and activation states rather than a uniform phenotype. Based on transcriptomic and functional profiling, CAFs are commonly classified into three major subtypes: myofibroblastic CAFs (myCAFs), characterized by high α-SMA expression and extracellular matrix remodeling; inflammatory CAFs (iCAFs), which secrete high levels of pro-inflammatory cytokines and chemokines; and antigen-presenting CAFs (apCAFs), which express major histocompatibility complex class II molecules and may modulate immune responses [19,20,21,22,23]. Depending on the nature, strength, and duration of tumor-derived stimuli, stromal cells may acquire partial, transient, or alternative activation states that do not necessarily fulfill all molecular or functional criteria of fully differentiated CAFs [24]. The origin of CAFs remains controversial since multiple cellular origins have been proposed, including resident fibroblasts, smooth muscle cells, pericytes, and mesenchymal stromal cells (MSCs), which can acquire a CAF-like phenotype upon exposure to tumor-derived signals [23,25,26,27]. Among MSCs, human adipose stromal cells (hASCs) are of particular relevance in regenerative medicine due to their abundance, plasticity, and multipotency [28,29]. Known inducers of MSC-to-CAF transdifferentiation include transforming growth factor beta 1 (TGF-β1), interleukins such as IL-6 and IL-8, tumor necrosis factor alpha (TNF-α), the CXCR4/CXCL12 axis, and extracellular vesicles carrying microRNAs and other bioactive molecules [30,31,32,33]. These stimuli converge on key signaling pathways such as SMAD, signal transducer and activator of transcription 3 (STAT3), nuclear factor kappa B (NF-κB), and Notch, ultimately leading to the upregulation of CAF-associated markers, which are commonly used but not exclusive indicators of CAF-like phenotypes, including alpha-smooth muscle actin (α-SMA), fibroblast activation protein (FAP), tenascin-C (TNC), secreted protein acidic and rich in cysteine (SPARC), vimentin (VIM), and tissue inhibitor of metalloproteinases 1 (TIMP1) [27,34,35,36,37].

Given that pro-inflammatory cytokines such as IL-1α, IL-1β, IL-6, and IL-8 are abundant in untreated HNSCC supernatants and remain detectable after HHP, it is important to assess whether these residuals can induce a CAF-like phenotype in surrounding stromal cells. Such an effect would represent a potential oncological risk for the reimplantation of HHP-treated grafts. In the present study, we examined whether hASCs exposed to conditioned media from untreated (0 MPa) or HHP-treated (300 MPa) HNSCC cell lines undergo a phenotypic and functional shift toward CAF-like cells. In addition, tumor-conditioned medium (TCM) with an extended conditioning period was included as a supplementary group to assess whether prolonged exposure to tumor-derived factors could enhance CAF conversion. TGF-β1 stimulation, a well-established inducer of CAF activation, served as a positive control in our study. Using a combination of morphological analysis, mRNA and protein quantification of CAF markers, and cytokine profiling, we aimed to determine whether HHP-treated tumor supernatants possess the capacity to induce a tumor-promoting stromal phenotype. This approach provides important insights into the oncological safety of HHP for the autologous reimplantation of tumor-infiltrated cartilage and advances our understanding of MSC-to-CAF conversion under tumor-derived stimuli.

## 2. Materials and Methods

### 2.1. Isolation and Cultivation of hASC

Subcutaneous adipose tissue was obtained from eight healthy donors undergoing elective procedures such as abdominoplasty, autologous fat transfer, or liposuction at the department for plastic surgery, University Hospital RWTH Aachen. Importantly, none of the samples were derived from tumor-associated tissue, all specimens originated from non-pathological surgical indications. All donors were informed about the use of their tissue for research purposes and provided written consent. The study was approved by the local Ethics Committee (EK163/07).

Adipose tissue was processed for isolation of hASCs as previously described [38]. Briefly, adipose tissue was minced and digested in a collagenase solution (0.2% collagenase I in PBS) for 45 min at 37 °C. The digested suspension was filtered through a 250 µm nylon mesh (Neolab, Heidelberg, Germany). Mature adipocytes, oil, and cell debris were separated from the stromal vascular fraction by centrifugation at 400× *g* for 10 min. The cell sediment was resuspended in Dulbecco’s modified Eagle medium (DMEM)/Ham’s F12 (1:1) (Pan Biotech, Aidenbach, Germany) supplemented with penicillin and streptomycin (P/S; Sigma-Aldrich, St. Louis, MO, USA; each at 100 U/mL), 0.1% bFGF (Peprotech, Cranbury, NJ, USA), 200 µM ascorbic acid, 10% fetal bovine serum (FBS, Pan Biotech, Aidenbach, Germany). Primary cells were expanded until they reached approximately 90% confluence. From passage 1 onward, the cells were cultured in DMEM/Ham’s F12 (1:1) supplemented with 10% FBS only. Cultures were maintained at 37 °C in a humidified atmosphere containing 5% CO_2_, and passages two to five were used for subsequent experiments.

HASCs were isolated from eight independent healthy donors; however, due to limited cell availability and assay-specific cell number requirements, not all experiments could be performed with hASCs from all donors. Subsets of donors were therefore used, while key findings were consistently reproduced across multiple independent donors.

### 2.2. HNSCC Cell Line Cultivation

The head and neck squamous cell carcinoma cell lines HNSCC16 (larynx P1 M1) and UTSCC14 (tongue, RRID:CVCL_7810) were utilized in this study. The primary tumor cell line HNSCC16 was established by the research group Schoenwalder et al. [39] and authenticated by STR profiling (against the respective patient tumor and patient-derived xenograft (PDX)). Both cell lines were human papillomavirus (HPV) negative, and all experiments were performed with mycoplasma-free cells.

Cancer cells were grown in DMEM/Ham’s F12 (1:1) (Pan Biotech, Aidenbach, Germany) supplemented with 10% FBS (Pan Biotech, Aidenbach, Germany) and 1% P/S (Sigma-Aldrich, St. Louis, MO, USA) and cultured in 75-cm^2^ culture flasks at 37 °C with 5% CO_2_ in a humified incubator. The medium was replaced every 2–3 days. Upon reaching a confluence of 80–90%, cells were washed with Dulbecco’s Phosphate-Buffered Saline (DPBS, Gibco, Waltham, MA, USA), detached with 0.25% Trypsin-ethylenediaminetetraacetic acid (EDTA, Pan Biotech, Aidenbach, Germany), and seeded in new flasks or treatment wells.

For the experiments DMEM/Ham’s F12 (1:1) supplemented with 1% Insulin-Transferrin-Selenium (ITS, Gibco, Waltham, MA, USA) and 1% P/S was used to eliminate interfering effects of FBS and is labeled as serum-free medium or control (CTRL) medium in this study.

### 2.3. High Hydrostatic Pressure Treatment and Preparation of Conditioned Medium

CM was prepared from HNSCC cells and subsequently used for hASC stimulation experiments. Two different types of CM were generated: (i) high hydrostatic pressure–treated conditioned medium (HHP-CM) and (ii) tumor-conditioned medium (TCM).

For HHP-CM, 1 × 10^6^ cells (HNSCC16 or UTSCC14) were suspended in serum-free medium (DMEM/F12 supplemented with 1% ITS and 1% P/S) and transferred into 1.8 mL cryotubes (Thermo Fisher Scientific, Waltham, MA, USA). Cryotubes were centrifuged at 120× *g* for 8 min, completely filled with serum-free medium, and carefully sealed to avoid air bubbles. To prevent leakage, tubes were wrapped with Parafilm M (Pechiney Plastic Packaging Inc., Stamford, CT, USA) and placed into water-filled, air-free centrifuge tubes, which were then positioned in the glycol-filled chamber of the high hydrostatic pressure device (Dustec Hochdrucktechnik GmbH, Wismar, Germany). Cells were subjected to HHP treatment at 300 MPa for 10 min at 20 °C or left untreated for reference samples (0 MPa). Following treatment, cryotubes were centrifuged again and incubated for 1 h at 37 °C to allow additional cytokine release from pressure-damaged cells and cytokine secretion from viable control cells (0 MPa). The resulting supernatants (conditioned media) were collected, transferred into new cryotubes, and stored at –80 °C until further use. Before application to hASC cultures, all HHP-CM samples were sterilized by filtration through 0.2 µm syringe filters (Corning, Inc., Corning, NY, USA).

For TCM, ~80% confluent T75 flasks of HNSCC16 or UTSCC14 cells were cultured in 10 mL serum-free medium for 24 h. The supernatant was collected, centrifuged at 3000× *g* to remove cell debris, sterilized through a 0.2 µm filter, and stored at –80 °C until use. The longer incubation period with a higher number of viable cells was chosen to allow sufficient accumulation of tumor-derived soluble factors and cytokines, providing a representative model of tumor-conditioned medium.

### 2.4. Exposure of hASC to Conditioned Medium

ASC were seeded either in a 6-well round bottom plate (Avantor, Inc., Radnor, PA, USA) at a density of 1 × 10^5^ cells for gene expression analysis and Western blot analysis, or in a 12-well glass bottom fibronectin-coated plate (Corning, Corning, NY, USA) at a density of 0.2 × 10^5^ cells for immunostaining. Cells were seeded in DMEM/F12 supplemented with 10% FBS and allowed to attach overnight. Culture medium was then replaced by serum-free medium for 24 h. Finally, hASCs were exposed to CM or control medium for 96 h, including a medium change at 48 h. Serum-free medium served as a negative control and serum-free medium supplemented with TGF-β1 (10 ng/mL; Peprotech, Cranbury, NJ, USA) was used as a positive control. On a 6-well plate, 2 mL of CM were added per well, whereas on a 12-well plate, 0.75 mL were used per well. All approaches were supplemented with 100 nM Dexamethasone (Peprotech, Cranbury, NJ, USA) throughout the incubation period to maintain ASC viability and phenotypic stability during prolonged serum-free culture. Low-dose dexamethasone preserves MSC stemness and reduces culture-induced drift [40]. Importantly, dexamethasone was applied uniformly across all conditions to avoid systematic bias and enable relative comparison of CAF-related responses to tumor-derived conditioned media. Finally, after 96 h of incubation, the spent medium was collected and frozen at −80 °C for subsequent cytokine analysis.

For subsequent use, cells were washed and either harvested for quantitative real time (qRT)-PCR, Western blot or ELISA analysis, or fixed for immunostaining.

### 2.5. Quantitative Real-Time PCR Analysis

To analyze the gene expression profile of CAF-associated markers, qRT-PCR was performed. Following exposure of hASCs to CM, total RNA was isolated using the RNeasy Micro Kit (Qiagen GmbH, Hilden, Germany) including on-column DNase digestion according to the manufacturer’s instructions. RNA concentration and purity were assessed with a NanoDrop spectrophotometer (Thermo Fisher Scientific, Waltham, MA, USA). Complementary DNA (cDNA) was synthesized from the isolated RNA using the Maxima First Strand cDNA Synthesis Kit (Thermo Fisher Scientific, Waltham, MA, USA).

The qRT-PCR was conducted using SYBR™ Green chemistry (Thermo Fisher Scientific, Waltham, MA, USA) with 10 ng cDNA template per reaction. Primer sets by Sigma-Aldrich were used for CAF-associated genes including *α-SMA*, *FAP*, *TNC*, *SPARC*, *TIMP1*, and *VIM*. Glyceraldehyde-3-phosphate dehydrogenase (GAPDH) served as the internal reference gene. The qRT-PCR system-integrated StepOne™ Software (version 2.3, Life Technologies, Carlsbad, NJ, USA) was used for evaluation. Relative mRNA expression levels were calculated using the 2^–ΔΔCt^ method.

### 2.6. Immunofluorescence Staining

Indirect immunostaining was performed to evaluate the expression of CAF-associated proteins. HASCs were cultured for 96 h with CM (HHP-CM or TCM) or control medium (serum-free medium or with TGF-β1). Cells were seeded at a density of 2 × 10^4^ cells on UV-sterilized glass coverslips coated with fibronectin and placed in 12-well plates. After incubation, cells were fixed with 4% paraformaldehyde (PFA; Otto Fischar GmbH & Co. KG, Saarbrücken, Germany) for 10 min at 37 °C, washed with DPBS, and stored in DPBS containing 1% P/S until further processing.

Blocking was performed with 5% bovine serum albumin (BSA, Merck KGaA, Darmstadt Germany) and 1% goat serum (Invitrogen, Carlsbad, NJ, USA) in PBS for 45 min at room temperature (RT). Primary antibodies were diluted in 1% BSA in PBS-T (PBS with 0.1% Tween-20 (Merck KGaA, Darmstadt, Germany)) and applied as follows: α-SMA (mouse monoclonal, 1:200, 1 h at RT; Abcam, Cambridge, UK), FAP (rabbit monoclonal, 1:200, overnight at 4 °C; Cell Signaling Technology, Danvers, MA, USA), and TNC (mouse monoclonal, 1:200, 1 h at RT; Invitrogen, Carlsbad, NJ, USA). After washing, secondary antibodies diluted in 1% BSA in PBS-T were applied for 1 h at RT: Alexa Fluor 594 goat anti-mouse IgG (1:400; Invitrogen, Carlsbad, NJ, USA) and Alexa Fluor 488 goat anti-rabbit IgG (1:400; Invitrogen, Carlsbad, NJ, USA). Nuclei were counterstained with Hoechst reagent (0.5 µg/mL; Sigma-Aldrich, St. Louis, MO, USA) for 5 min at RT. Following final washes, wells were covered with PBS containing 1% P/S and imaged using a fluorescence microscope (Carl Zeiss AG, Oberkochen, Germany) at 10× or 20× magnification, respectively.

For α-SMA and FAP, the mean fluorescence intensity (MFI) was quantified on a per-cell basis by segmenting individual cells and determining the MFI within each single-cell mask. The MFI of the unstimulated control, including its standard deviation (SD) served as reference threshold for determination of positively marked cells. The proportion of positive cells was then calculated as the percentage of stained cells relative to the total number of nuclei in ten independent fields per condition, and values were normalized to the respective negative control. At least 200 cells per condition and per donor were analyzed. For TNC, mean fluorescence intensity per cell was quantified and normalized to the corresponding negative control. Image analysis was performed with ImageJ (version 1.54f, National Institutes of Health, Bethesda, MD, USA). Each condition was analyzed in duplicate for each marker.

### 2.7. Microscopy and Analysis of Cell Numbers

Morphological changes in hASCs were documented after 96 h of incubation using a Leica light microscope (Leica, Wetzlar, Germany). Immunofluorescence images were acquired with a Zeiss Axio Observer microscope equipped with a fluorescence Colibri5 light source providing different excitation wave lengths, an LED light source, an Axiocam 503 Mono, and the corresponding Zen 3.8 software (Carl Zeiss AG, Oberkochen, Germany) at 10× or 20× magnification following immunofluorescence staining. Using the optimally adjusted setups for the dyes employed in this study, all experimental conditions within and between donors were imaged under identical settings, including exposure times, to ensure comparable data aquisition. A negative control containing only the secondary antibody was included in each staining. Merged images are presented in the main figures, while the individual fluorescence channels are shown in the Supplementary Materials (Supplementary Figures S1 and S2).

Proliferation data were derived from the DAPI channel of the immunofluorescence images. For quantification, nuclei were counted in ten randomly selected fields per condition (10× magnification). For morphological analysis, cell area and aspect ratio were quantified using ImageJ software. Individual cells were manually segmented based on cytoplasmic staining, and cell area was measured following spatial calibration. Cell elongation was assessed by calculating the aspect ratio as the ratio of the major to the minor cell axis. For each condition, measurements were performed on cells from three randomly selected fields per donor (20× magnification).

### 2.8. Western Blot Analysis

For protein isolation, hASCs cultured under the respective experimental conditions were washed with cold DPBS and lysed directly on the culture plates using RIPA buffer supplemented with protease-inhibitor (Roche, Basel, Switzerland). Cells were detached by scraping, and the lysates were collected and incubated on ice for 30 min with intermittent mixing. Subsequently, samples were centrifuged at 14,000× *g* for 15 min at 4 °C to remove cell debris. The supernatant was transferred to fresh tubes, and protein concentrations were determined using a BCA assay (Thermo Fisher Scientifc, Waltham, MA, USA). All samples were adjusted to equal protein concentrations prior to further processing.

For SDS-PAGE, 20 µg of total protein per condition were loaded onto a 10-well TGX Stain-Free™ gel (Bio-Rad Laboratories, Hercules, UK). Each protein sample (15 µL) was mixed with 4× Laemmli loading buffer (Bio-Rad Laboratories, Hercules, UK) supplemented with β-mercaptoethanol (Merck KGaA, Darmstadt, Germany; 1:9, β-mercaptoethanol:loading buffer) in a ratio of 3:1 (sample:loading dye) and loaded onto the gel. Precision Plus Protein™ All Blue Prestained Standards (Bio-Rad Laboratories, Hercules, UK) served as molecular weight reference. Electrophoresis was carried out at 120 V for approximately 45 min.

Proteins were transferred onto LF-PVDF membranes using the Trans-Blot Turbo Transfer System (Bio-Rad Laboratories, Hercules, UK) with the Mixed MW (Turbo) program (7 min, 1.3 A, 25 V). Membranes were blocked for 1 h at RT in PBS containing 5% (*w*/*w*) BSA and 0.05% (*v*/*v*) Tween-20. α-SMA antibody (1:1000, mouse monoclonal, Abcam, Cambridge, UK) was diluted in TBS-T and incubated for 1 h at RT. After three washes in TBS containing 0.05% Tween-20, membranes were incubated for 1 h at RT with fluorophore-conjugated secondary antibodies (StarBright^®^ goat anti-mouse IgG, 1:5000, Bio-Rad Laboratories, Hercules, UK).

Fluorescent signals were acquired using the ChemiDoc MP Imaging System (Bio-Rad Laboratories, Hercules, UK) with the appropriate emission channels. Stain-Free technology was applied to normalize for total protein transfer, serving as a loading control. Band intensities were quantified using Image Lab software (Version 6.1.0 build 7, Bio-Rad Laboratories, Hercules, UK). Uncropped Western blot images and stain free images are provided in the Supplementary Materials (Supplementary Figure S3).

### 2.9. Enzyme-Linked Immunosorbent Assay

Determination of FAP concentration was performed on cell lysates using the DuoSet Human FAP enzyme-linked immunosorbent assay (ELISA) kit (R&D Systems, Minneapolis, MN, USA) according to the manufacturer’s instructions. For each sample, 1 µg of total protein was diluted in the sample buffer supplied with the kit, and both samples and standards were pipetted in duplicate into pre-coated wells. Following incubation with the detection antibody and subsequent addition of the streptavidin–HRP conjugate, color development was achieved using the substrate solution provided in the kit.

Absorbance was measured at 450 nm using a microplate reader (Molecular Devices, San Jose, CA, USA). FAP concentrations were determined from a standard curve generated with recombinant FAP standards using a four-parameter logistic (4-PL) curve fit.

### 2.10. Cytokine Analyses

The Human Cytokine Array C3 (RayBiotech Inc., Norcross, GA, USA) Dot blot assay was used as an initial screening method to analyze the secretion profile of 42 cytokines, including key pro-inflammatory cytokines, chemokines, and growth factors. The assay was performed on thawed HHP-CM, TCM or control medium after incubation with hASCs. The assay was conducted according to the manufacturer’s instructions. Labeled proteins were visualized via enhanced chemiluminescence using detection buffer and X-ray film exposure. Cytokines appeared as dots of varying intensity and size.

For quantitative analysis, selected cytokines identified in the screening assay, MCP-1, IL-6, IL-8, MCP-3, CCL5, and CXCL5 were measured using the LEGENDplex™ Human Custom Panel (BioLegend, San Diego, CA, USA). HHP-CM, TCM and control medium were analyzed both prior to their addition to hASCs and after 48 h of ASC cultivation. Briefly, CM was incubated with capture beads overnight at 4 °C on an orbital shaker. Detection antibodies were then added, and samples were incubated for 1 h at RT. After washing, bead fluorescence was measured on a FACSCanto™ flow cytometer (BD Biosciences, Franklin Lakes, NJ, USA). Cytokine concentrations were calculated from standard curves using the LEGENDplex data analysis software (version 2023-02-15, BioLegend, San Diego, CA, USA). To account for baseline cytokine levels, concentrations measured in CM before ASC incubation were subtracted from the corresponding post-incubation values. The cytokine concentration then was normalized to the cell number determined before in the proliferation analysis (see Section 2.7). All measurements were performed in duplicate.

### 2.11. Statistical Analysis

Statistical analysis was performed using GraphPad PRISM software, version 8.0.1 (GraphPad Software, Boston, MA, USA). A *p*-value < 0.05 was considered statistically significant. Each individual hASC donor and each experimental run were considered independent biological replicates for statistical analysis. As different hASC donors were assigned to independent treatment conditions, comparisons across more than two groups were performed using ordinary one-way ANOVA for normally distributed data. In cases of non-normal distribution, the Kruskal–Wallis test was applied.

Predefined post hoc pairwise comparisons were conducted to assess (i) induction relative to baseline by comparing the negative control with all other experimental conditions (TGF-β1, TCM, 0 MPa-CM, and 300 MPa-CM) and (ii) the effect of HHP treatment by comparing TCM and 0 MPa-CM to 300 MPa-CM. For parametric analyses, multiple comparisons were corrected using the Holm–Šídák method, whereas for non-parametric analyses, Dunn’s multiple comparisons test was applied.

## 3. Results

### 3.1. Morphological Characterization of hASCs After Incubation with Conditioned Media

Morphological alterations of hASCs were analyzed after incubation with the respective conditioned media to identify potential phenotypic changes indicative for CAF-like transdifferentiation.

**Figure 1:**
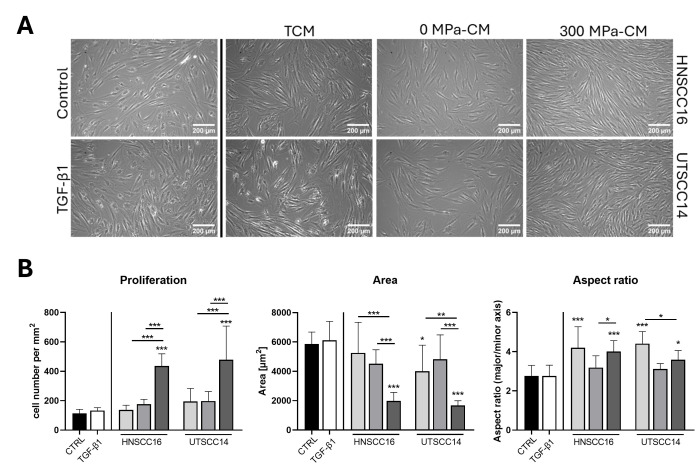
Morphology and proliferation of human adipose stromal cells (hASCs) after 96 h incubation with conditioned media (CM). (**A**) HASCs were incubated for 96 h with tumor-conditioned medium (TCM), 0 MPa-CM, or 300 MPa-CM derived from HNSCC16 or UTSCC14 cells, TGF-β1 (10 ng/mL) or serum-free control medium, respectively. Representative phase-contrast images of one out of eight donors are shown. Scale bar: 200 µm. (**B**) Proliferation and morphological quantification analysis of hASC after cultivation with CM or control medium for 96 h. For proliferation analysis, cell numbers were quantified via Hoechst fluorescence from three different donors (mean ± SD, n = 3). Morphological analysis was performed using ImageJ. Statistical significance was assessed using predefined post hoc comparisons following one-way ANOVA. Differences were evaluated relative to the control group or between specific treatment conditions as indicated in the figures. Statistically significant differences are indicated by asterisks (* *p* < 0.05, ** *p* < 0.01, *** *p* < 0.001). CTRL = control.

After 96 h of stimulation with the respective conditioned media, cells were examined by light microscopy to assess morphological alterations (Figure 1A). HASCs cultured in serum-free control medium predominantly displayed a spread, polygonal morphology typical for undifferentiated hASCs, although some cells appeared slightly elongated and spindle-shaped. Cells treated with TGF-β1 also exhibited mainly spread cell bodies without marked elongation. In contrast, hASCs incubated with TCM or 0 MPa-CM of HNSCC16 and UTSCC14 showed a predominantly spindle-shaped morphology. Cells exposed to 300 MPa-CM derived from HNSCC16 or UTSCC14 displayed elongated, fibroblast-like shapes forming strand-like growth patterns.

After 96 h, exposure to 300 MPa-CM resulted in a significant increase in hASC cell numbers in both HNSCC16- and UTSCC14-derived conditions compared to the control, TCM, and 0 MPa-CM groups (Figure 1B; 2.5–3.8-fold for HNSCC16 and 2.4–4.2-fold for UTSCC14).

Quantification of cell area (Figure 1B) revealed comparable values in control and TGF-β1-treated hASCs. In both HNSCC16- and UTSCC14-derived conditions, cell area was significantly reduced following exposure to 300 MPa-CM compared to the control, TCM, and 0 MPa-CM conditions. The aspect ratio (Figure 1B), defined as the ratio of the major to the minor cell axis, was used to quantify cell elongation. Control and TGF-β1–treated hASCs showed comparable aspect ratio values. In both HNSCC16- and UTSCC14-derived conditions, aspect ratio values were significantly increased following exposure to TCM and 300 MPa-CM compared to the control. In HNSCC16-derived conditions, 300 MPa-CM–treated hASCs exhibited a significantly higher aspect ratio than the corresponding 0 MPa-CM condition, whereas in UTSCC14-derived conditions, TCM-treated cells showed significantly higher aspect ratio values compared to 300 MPa-CM.

### 3.2. CAF Marker Gene Expression in hASCs After Incubation with Conditioned Media

**Figure 2:**
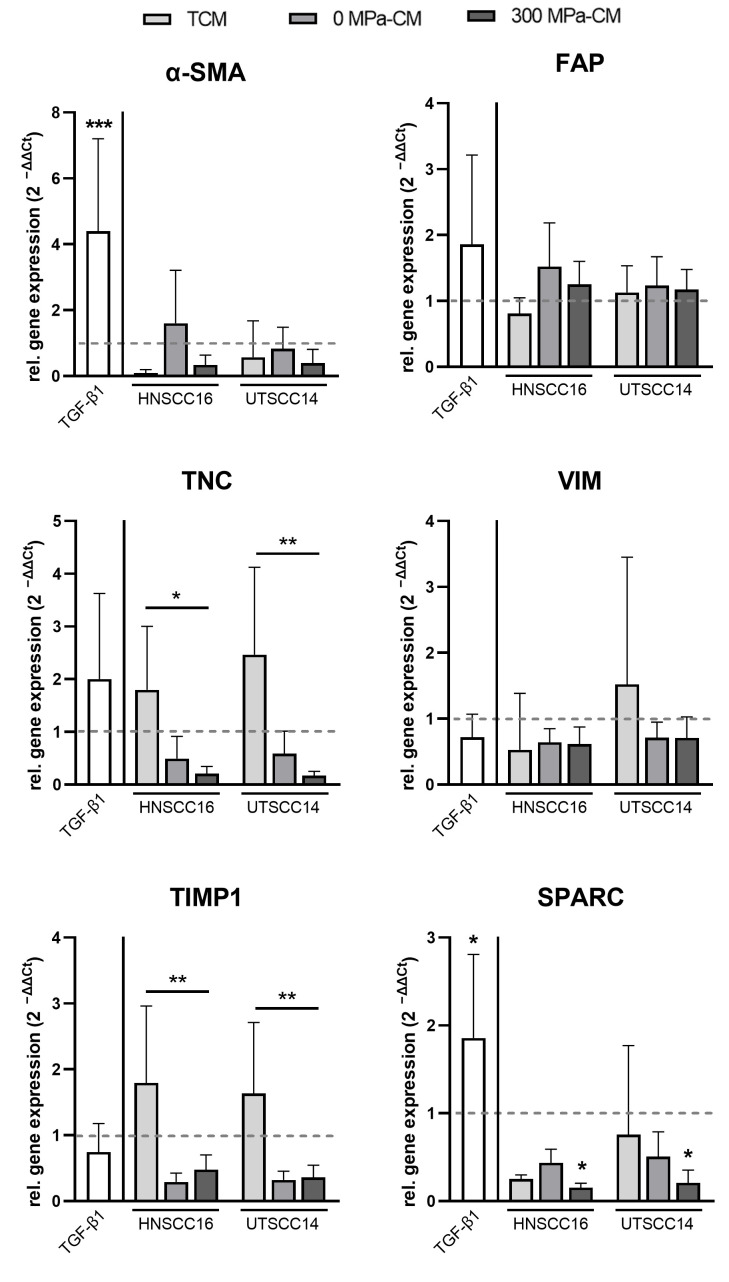
Relative mRNA expression of cancer-associated fibroblast (CAF) markers in human adipose stromal cells (hASCs) after stimulation with conditioned media (CM). HASCs were incubated for 96 h with tumor-conditioned medium (TCM), 0 MPa-CM, or 300 MPa-CM derived from HNSCC16 or UTSCC14 cells, TGF-β1 (10 ng/mL) or serum-free control medium, respectively. The expression of α-SMA, FAP, TNC, VIM, TIMP1, and SPARC was analyzed by quantitative real time-PCR. Evaluation was performed using the 2^−ΔΔCt^ method. Data are presented as relative expression normalized to Glyceraldehyde-3-phosphate dehydrogenase (GAPDH; mean ± SD, n = 7–8 independent donors) and shown as fold change compared to the control (set to 1; illustrated as dashed lines). Statistical significance was assessed using predefined post hoc comparisons following one-way ANOVA or Kruskal–Wallis testing. Differences were evaluated relative to the control group or between specific treatment conditions as indicated in the figures. Statistically significant differences are indicated by asterisks (* *p* < 0.05, ** *p* < 0.01, *** *p* < 0.001).

To investigate a potential CAF-like transdifferentiation of hASCs under the influence of different conditioned media, the relative mRNA expression levels of several representative markers were quantified by qRT-PCR (Figure 2). Since no single marker exclusively defines CAFs, a panel including α-SMA, FAP, TNC, TIMP1, VIM, and SPARC was selected to capture distinct aspects of fibroblast activation and matrix remodeling. For all analyses, expression values of CM-treated hASCs were normalized to the serum-free medium control. This control was defined as 1, and all other conditions were reported as fold changes relative to it.

Expression of the activation marker α-SMA remained low in hASCs exposed to all conditioned media, except for 0 MPa-CM from HNSCC16, which showed a slight upregulation (1.6-fold). TGF-β1 stimulation led to a significant upregulation of α-SMA compared to the control (4.4-fold). FAP expression remained at baseline levels across all CM conditions, although a modest increase (1.5-fold) was detected in the 0 MPa-CM condition of HNSCC16. In contrast, TGF-β1 treatment resulted in only a minor, non-significant increase in FAP expression (1.8-fold). TNC expression was upregulated following stimulation with TCM from both cell lines (1.8- and 2.5-fold, respectively) and was downregulated in response to 0 MPa- and 300 MPa-CM compared to the control. For both HNSCC16 and UTSCC14, TNC expression in the 300 MPa-CM condition was significantly lower than in the corresponding TCM. TGF-β1 stimulation resulted in an approximately twofold increase in TNC expression. VIM was constitutively expressed at slightly lower levels than the control across most conditions. Only TCM from UTSCC14 induced a minor, non-significant increase in VIM expression (1.5-fold) with high variability across donors. TIMP1 expression wasupregulated in response to TCM from both cell lines (16–1.8-fold). In contrast, 0 MPa-CM and 300 MPa-CM resulted in expression levels lower than the control. Accordingly, comparison of TCM to 300 MPa-CM revealed a significant downregulation of TIMP1 expression in both cell lines in response to the HHP-treated CM. SPARC expression was decreased in response to all conditioned media conditions, with a statistically significant reduction observed in both cell lines in the 300 MPa-CM condition relative to the control. In contrast, TGF-β1 stimulation led to a significantly higher SPARC expression than in the control (1.9-fold).

### 3.3. Protein Analysis of CAF-Associated Protein Expression in hASCs After Incubation with Conditioned Media

Among CAF markers, α-SMA and FAP are regarded as the most prominent CAF-associated proteins and were therefore analyzed by immunostaining. In addition, the extracellular matrix glycoprotein TNC, which is closely linked to CAF conversion and tissue remodeling, was included to provide a more comprehensive phenotypic characterization.

**Figure 3:**
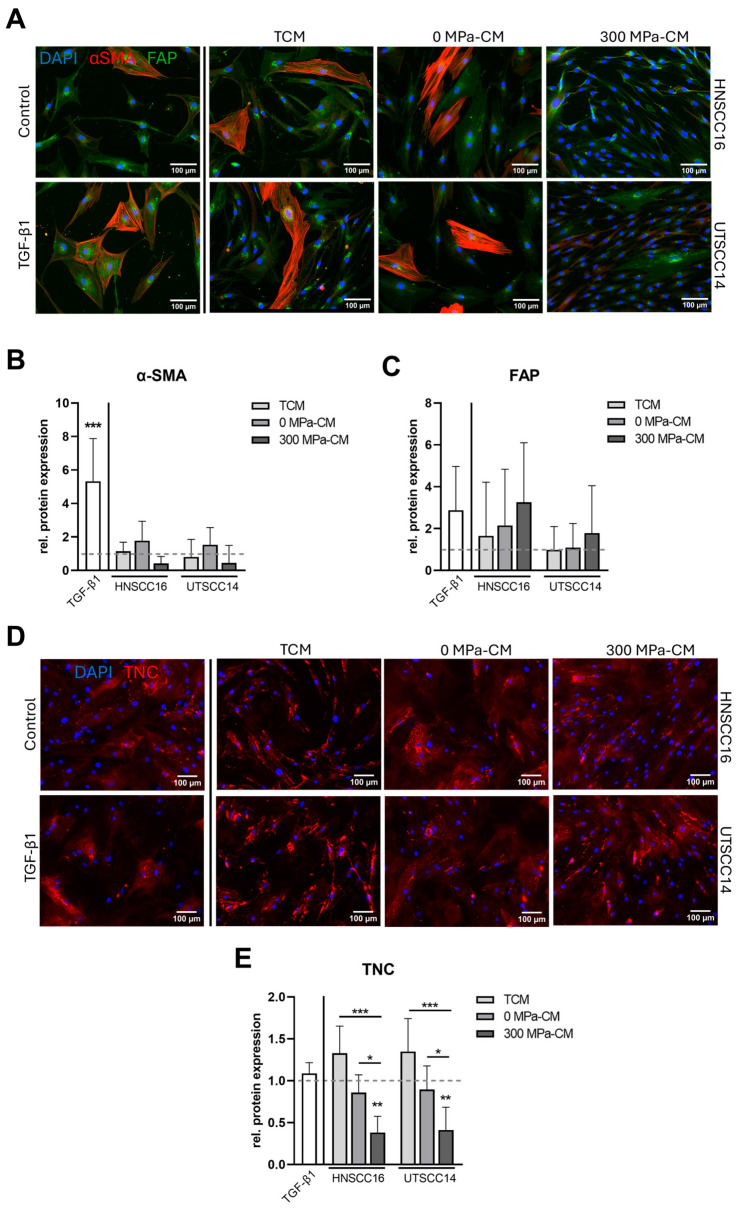
Immunofluorescence analysis of cancer-associated fibroblast (CAF) markers in stimulated human adipose stromal cells (hASCs). HASCs were incubated for 96 h with tumor-conditioned medium (TCM), 0 MPa-CM, or 300 MPa-CM derived from HNSCC16 or UTSCC14 cells, TGF-β1 (10 ng/mL), or serum-free control medium, respectively. (**A**) Representative immunofluorescence images showing staining of CAF markers α-SMA (red, intracellular) and FAP (green, membrane-associated) with nuclear counterstaining using Hoechst (blue). Scale bar: 100 µm. (**B,C**) Quantification of relative α-SMA (**B**) and FAP (**C**) fluorescence intensity across all samples (mean ± SD, n = 4 independent donors, each analyzed in duplicate from two independent experiments). Data are presented as relative protein expression normalized to the serum-free control (set to 1; illustrated as dashed lines). (**D**) Representative immunostaining of TNC (red) and Hoechst (blue) after 96 h of stimulation. Scale bar: 100 µm. (**E**) Quantification of TNC fluorescence intensity (mean ± SD, n = 3 independent donors, each analyzed in duplicate from two independent experiments). Results are shown as relative expression normalized to the serum-free control (set to 1). Statistical significance was assessed using predefined post hoc comparisons following one-way ANOVA or Kruskal–Wallis testing. Differences were evaluated relative to the control group or between specific treatment conditions as indicated in the figures. Statistically significant differences are indicated by asterisks (* *p* < 0.05, ** *p* < 0.01, *** *p* < 0.001).

Staining of α-SMA (Figure 3A) revealed that hASCs treated with 300 MPa-CM dis-played markedly lower signal intensities compared to all other experimental groups. Quantitative analysis (Figure 3B) confirmed significantly higher α-SMA intensities in the TGF-β1-treated group (5.3-fold) compared to the control. In contrast, 0 MPa-CM from both cell lines induced only a slight increase in α-SMA intensity (1.5–1.8-fold). Notably, α-SMA expression in the 300 MPa-CM condition was markedly lower than in the control and all other conditions. Staining of FAP (Figure 3A,C) revealed a 2.9-fold increase in protein expression following TGF-β1 stimulation. In hASCs treated with CM from HNSCC16, both TCM and 0 MPa-CM induced 1.7–2.1-fold higher mean fluorescence intensities compared to the control, whereas 300 MPa-CM resulted in a 3.3-fold increase in FAP expression, although accompanied by high variability. In contrast, 300 MPa-CM from UTSCC14 induced only a minor increase in FAP expression, while TCM and 0 MPa-CM from UTSCC14 remained at baseline levels. Notably, no statistically significant differences were detected between the CM-treated groups. TNC staining (Figure 3D,E) showed a slight increase in signal intensity in hASCs incubated with TCM from both HNSCC cell lines (1.3–1.4-fold) compared to the control, whereas TGF-β1 stimulation did not induce TNC expression. Cells exposed to 0 MPa-CM exhibited a slight reduction in TNC signal, whereas treatment with 300 MPa-CM resulted in a marked and statistically significant decrease in TNC expression compared to the control. Moreover, TNC levels were significantly reduced in both cell lines following 300 MPa-CM treatment compared to the respective TCM and 0 MPa-CM conditions.

**Figure 4:**
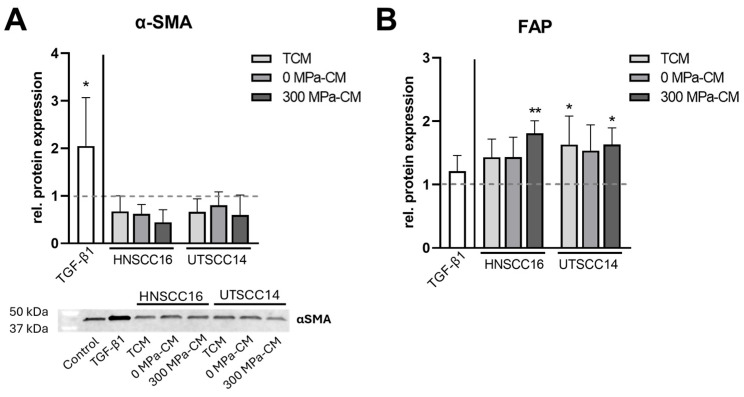
Protein expression analysis of cancer-associated fibroblast (CAF) markers in stimulated human adipose stromal cells (hASCs). HASCs were incubated for 96 h with tumor-conditioned medium (TCM), 0 MPa-CM, or 300 MPa-CM derived from HNSCC16 or UTSCC14 cells, TGF-β1 (10 ng/mL), or serum-free control medium, respectively. (**A**) α-SMA protein expression was determined by Western blot analysis. Representative Western blot membrane with protein bands of α-SMA following incubation with primary and secondary antibody is shown. Precision Plus Protein™ All Blue prestained standard served as molecular weight marker. Densitometric quantification of α-SMA protein expression was evaluated and total protein normalization was performed using Bio-Rad Stain-Free™ technology. Data represents n = 4 independent donors (mean ± SD). Results are presented as relative protein expression normalized to the serum-free control (set to 1; illustrated as dashed lines). (**B**) FAP levels were determined by ELISA in hASCs from six independent donors after 96 h of stimulation with the respective conditioned media. Results are presented as relative protein expression normalized to the serum-free control (set to 1; illustrated as dashed lines). Statistical significance was assessed using predefined post hoc comparisons following one-way ANOVA or Kruskal–Wallis testing. Differences were evaluated relative to the control group or between specific treatment conditions as indicated in the figures. Statistically significant differences are indicated by asterisks (* *p* < 0.05, ** *p* < 0.01).

In addition to the immunofluorescence analyses, α-SMA expression was examined by Western blot, and FAP levels were quantified using ELISA (Figure 4). Quantitative densitometric analysis of the Western blot, normalized to total protein, confirmed an approximately two-fold and statistically significant increase in α-SMA expression exclusively in the TGF-β1-treated samples compared to the control, whereas all other conditions remained slightly below baseline levels.

Quantification of FAP concentrations by ELISA revealed only minor variations across treatment groups. TGF-β1 induced a slight increase in FAP levels (1.2-fold) compared to the control. HASCs exposed to TCM from both HNSCC16 and UTSCC14 showed moderately elevated FAP concentrations (1.4–1.6-fold), comparable to those observed with 0 MPa-CM (1.4–1.5-fold). Treatment with 300 MPa-CM resulted in a 1.6–1.8-fold increase in FAP levels. Upregulation reached statistical significance in the TCM group of UTSCC14 and in the 300 MPa-CM conditions from both cell lines compared to the control; however, no statistically significant differences were observed between the treatment groups.

### 3.4. Cytokine Secretion Profile of hASCs After Incubation with Conditioned Media

To identify cytokines secreted by hASCs in response to conditioned media, a human cytokine antibody array was applied on culture supernatants collected after 96 h of incubation (Supplementary Figure S4A,B). This assay enabled the simultaneous detection of 42 cytokines, including key pro-inflammatory mediators, chemokines, and growth factors. Based on the resulting cytokine profiles, the most prominently regulated factors (MCP-1, IL-6, IL-8, CCL5, CXCL5, and MCP-3) were selected for further quantitative analysis using a bead-based multiplex assay.

The Dot blot membranes (Supplementary Figure S4A,B) revealed distinct cytokine secretion patterns depending on the applied conditioned medium. In the serum-free control, signals were mainly detected for MCP-1 and IL-6. In contrast, stimulation with TCM from HNSCC16 markedly enhanced the secretion of several cytokines, most prominently MCP-1, IL-6, IL-8, and CCL5. Additional signals for MCP-3 and CXCL5 were observed at 0 MPa-CM and 300 MPa-CM.

**Figure 5:**
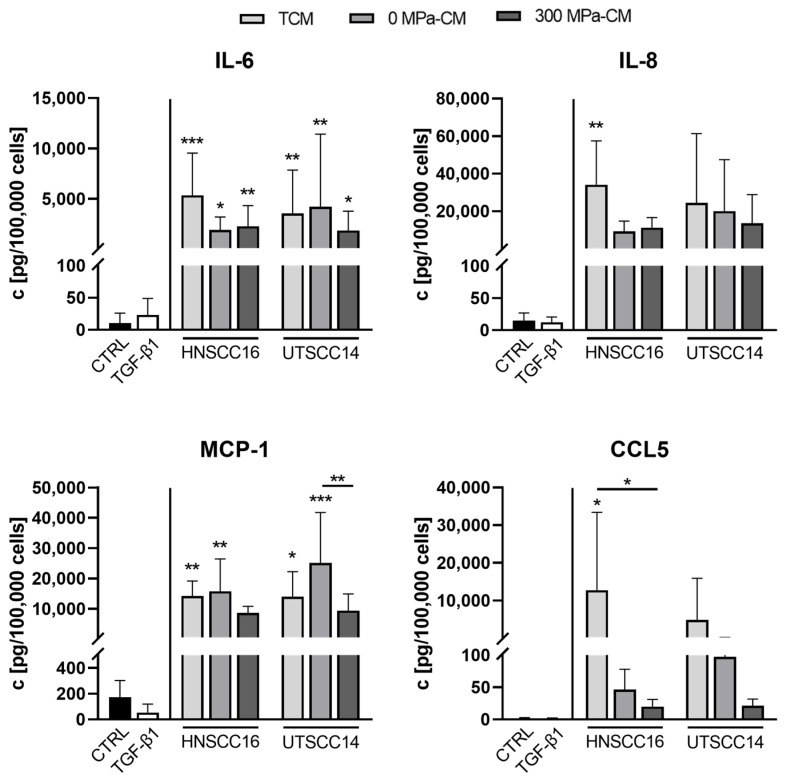
Quantification of cytokines secreted by human adipose stromal cells (hASCs) after incubation with conditioned media (CM). HASCs were incubated for 96 h with tumor-conditioned medium (TCM), 0 MPa-CM, or 300 MPa-CM derived from HNSCC16 or UTSCC14 cells, TGF-β1 (10 ng/mL), or serum-free control medium, respectively. After 48 h, the medium was replaced with fresh CM, and the supernatants were collected for cytokine analysis. Quantification of selected cytokines (IL-6, IL-8, MCP-1, CCL5) in hASC supernatants was determined by multiplex bead-based assay. Values represent the mean ± SD of n = 8 independent donors. Cytokine concentrations measured before and after incubation with hASCs were measured, and the difference (post–pre) normalized to 100,000 cells is shown. Statistical significance was assessed using predefined post hoc comparisons following one-way ANOVA or Kruskal–Wallis testing. Differences were evaluated relative to the control group or between specific treatment conditions as indicated in the figures. Statistically significant differences are indicated by asterisks (* *p* < 0.05, ** *p* < 0.01, *** *p* < 0.001). c = concentration; CTRL = control.

Quantitative analysis of selected cytokines (Figure 5) was then performed to validate and compare the secretion profiles of hASCs under the different experimental conditions. Cytokine levels were measured both in the respective conditioned media prior to hASC incubation and in the supernatants collected after cultivation, thereby capturing the hASC response to the applied stimuli. For each condition, cytokine concentrations detected in the original CM were subtracted from the values obtained after hASC cultivation to isolate hASC-derived secretion. Finally, all cytokine concentrations were normalized to the total cell number determined from Hoechst-stained images to account for the varying proliferation rates observed between treatments.

Overall, both the negative control and the TGF-β1-treated condition showed only minimal cytokine secretion, whereas hASCs exposed to TCM, 0 MPa-CM, and 300 MPa-CM from both tumor cell lines exhibited markedly elevated cytokine levels.

IL-6 concentrations were significantly increased in all conditioned media groups compared to the control; however, no significant differences in IL-6 secretion were observed between the CM-treated groups.

IL-8 levels in all CM conditions from both cell lines were higher compared to the control and TGF-β1 treatment; however, a statistically significant upregulation was observed only in the TCM condition of HNSCC16.

For MCP-1, hASCs stimulated with TCM and 0 MPa-CM from both HNSCC16 and UTSCC14 showed significantly higher MCP-1 concentrations compared to the control. Additionally, for UTSCC14, MCP-1 secretion following exposure to 300 MPa-CM was significantly reduced compared to the corresponding 0 MPa condition.

CCL5 secretion in the serum-free control and after TGF-β1 stimulation was nearly undetectable. In contrast, CCL5 levels were significantly increased after treatment with TCM from HNSCC16 and were significantly lower following exposure to 300 MPa-CM from the same cell line.

CXCL5 concentrations (Supplementary Figure S4C) were elevated in all CM groups compared to the serum-free control and TGF-β1 stimulation. Notably, CXCL5 levels in the 300 MPa-CM condition from UTSCC14 were significantly increased compared to the control. For MCP-3 (Supplementary Figure S4C), cytokine levels were higher in response to TCM, 0 MPa-CM, and 300 MPa-CM derived from HNSCC16 compared to the control.

## 4. Discussion

Oncological safety is crucial for autologous reconstruction following resection of cartilage-infiltrating head and neck tumors. Potential residual cytokines remaining after HHP treatment must be carefully evaluated, as they could affect stromal or immune cells, including mesenchymal stem cells within the tumor microenvironment. Since MSCs can differentiate into CAF-like cells under the influence of tumor-derived soluble factors, it is essential to exclude that cytokines released during HHP treatment could trigger a transformation into CAFs [41,42]. In this study, hASCs were chosen as a well-established model for mesenchymal stem cells involved in wound healing [43,44,45,46]. During tissue repair, these cells migrate into the reconstruction site and are exposed to local cytokines and growth factors, making them a relevant in vitro system to assess potential effects of HHP-treated tumor-derived mediators on stromal cell activation [47,48]. Accordingly, we examined whether conditioned media from HHP-treated HNSCC cells could induce a CAF-like phenotype in hASCs. By combining gene and protein expression, with cytokine analyses and morphological assessment, we aimed to evaluate the biological safety of HHP treatment for potential clinical applications. Fibroblasts can acquire a myofibroblast-like phenotype upon exposure to TGF-β1, and several studies further demonstrated that MSCs likewise undergo myofibroblast transdifferentiation with increased α-SMA production in response to TGF-β1 stimulation [49,50,51,52]. Accordingly, inhibition of the TGF-β1 pathway in hASCs has been proposed as a potential strategy to prevent acquiring a tumor-supportive phenotype and contributing to the tumor microenvironment [50]. In our present study, stimulation of hASCs with TGF-β1 also consistently induced a CAF-like phenotype, characterized by a pronounced upregulation of α-SMA and a moderate increase in FAP and TNC expression at mRNA and protein levels. This finding confirms the established role of TGF-β1 as a central regulator of fibroblast activation and myofibroblast transdifferentiation and validates the reliability of the experimental system applied in this study. In comparison, TCM and 0 MPa-CM from untreated HNSCC cells induced only isolated, modest changes in CAF marker expression, without indicating a consistent activation pattern. This mild response contrasts with findings by Werner et al., who reported a marked elevation of α-SMA expression at both the mRNA and protein levels in MSCs from human bone marrow after 24 and 48 h incubation with TCM from HCT8 colorectal cancer cells [53]. Remarkably, in their study, the TCM exerted an almost comparable effect on α-SMA induction as direct TGF-β1 stimulation, suggesting that tumor-derived soluble factors can strongly promote myofibroblast-like transdifferentiation of MSCs depending on the tumor type and its specific secretome composition [53]. The weaker effect observed in our HNSCC model may therefore reflect differences in cytokine abundance and composition among tumor entities and/or a comparatively lower TGF-β1 activity within HNSCC-derived CM, in line with our previous findings showing no relevant TGF-β1 abundance in any of the tested CM samples [13]. Additionally, we observed large interindividual variance across hASC donors that contributed to the modest overall effect sizes. Since primary cells exhibit donor-specific differences in cytokine responsiveness, proliferation, and differentiation potential, this biological heterogeneity must be considered when interpreting CAF marker expression and cytokine secretion data [54]. CM derived from HHP-treated HNSCC cells did not trigger any substantial CAF-associated response. Although hASCs treated with 300 MPa-CM showed an altered morphology with elongated, spindle-shaped cell bodies and reduced cell area, no further indications of CAF-like transdifferentiation were observed. Instead, most markers in gene and protein expression analyses showed a tendency toward downregulation, with significantly lower effects observed for 300 MPa-CM compared to either TCM or 0 MPa-CM. An exception was a modest increase in FAP levels detected by ELISA. As FAP is not an exclusive CAF marker and no concomitant upregulation of other CAF markers, particularly α-SMA, was observed, this does not indicate stable CAF-like transdifferentiation.

Notably, both HNSCC cell lines exhibited largely consistent response patterns across molecular, cytokine, and morphological readouts, indicating that the observed effects were not driven by cell line-specific properties but rather reflect a general response to HHP-treated versus untreated tumor-derived conditioned media.

Morphological changes observed in hASCs after exposure to 300 MPa-CM were accompanied by significantly increased proliferation rates. Importantly, morphological alteration of MSCs is not specific to CAF commitment and can result from transient cytoskeletal remodeling in response to non-differentiating stimuli, including changes in cell density, cytokine exposure, or culture conditions [55,56]. Factors that may be released from pressure-damaged cells and are potentially present in 300 MPa-CM, such as inflammatory cytokines, stress-associated mediators, or damage-associated molecular patterns (DAMPs), could modulate cell adhesion and actin dynamics via pathways regulating cytoskeletal organization without activating a CAF-associated transcriptional program [57,58]. Consistent with this notion, no concomitant upregulation of canonical CAF markers at the mRNA or protein level was observed, arguing against stable CAF-like transdifferentiation and suggesting that the morphological alterations reflect a transient activation or adaptive response rather than durable pro-fibrotic stromal reprogramming. In a previous study investigating the effects of HHP-CM on vital tumor cells, we observed that HNSCC16 cells treated with 0 MPa-CM exhibited significantly increased proliferation compared to serum-free controls, whereas treatment with 300 MPa-CM did not affect cell numbers [13]. This apparent discrepancy between tumor and stromal cell responses can likely be attributed to cell type–specific responses to the altered cytokine milieu following HHP treatment. Such proliferative responses are characteristic of early wound-healing–like activation states in MSCs, in which stromal progenitor cells transiently expand in response to tissue damage and inflammatory cues to support regeneration and repair [47,48,59].

Cytokine analyses provided further mechanistic insights into the response of hASCs to the different conditioned media. In this study, cytokine concentrations were measured both in the conditioned media before hASC stimulation and in the supernatants collected afterward. By subtracting the cytokine levels present in the original CM from those detected after cultivation, the values presented here represent the isolated cytokine secretion derived from hASCs in response to the respective stimuli. HASCs exposed to TCM secreted markedly higher levels of IL-6, IL-8, MCP-1, and CCL5 compared to both the negative and the TGF-β1 controls. IL-6 and IL-8 promote fibroblast activation, inflammation, proliferation, and secretion of matrix-remodeling enzymes, while MCP-1 recruits monocytes and macrophages that may further amplify pro-tumorigenic signaling through feedback cytokine loops [60,61]. CCL5, in particular, has been associated with myofibroblast-like transdifferentiation of tumor-activated hASCs and contributes to a self-reinforcing stromal activation cycle. Escobar et al. (2015) demonstrated that MSCs secreted higher levels of chemokines, including CCL5, when exposed to TCM from aggressive breast cancer cells compared to less aggressive ones, highlighting that the tumor’s cytokine profile critically shapes stromal behavior [62]. In line with these findings, CCL5 secretion in our study was only detectable in the TCM from both HNSCC cell lines, whereas it was absent in all HHP-treated CM. In response to 300 MPa-CM, significant lower cytokine levels of MCP-1, IL-8, and CCL5 were observed compared to TCM. This observation indicates that while HHP-treated CM can still induce a limited inflammatory response in hASCs, the magnitude of this effect is reduced compared to untreated tumor CM. Supernatants from HHP-treated tumor cells have previously been shown to contain high levels of IL-1α, IL-1β, IL-6 and IL-8, potent mediators of inflammatory responses [13]. However, despite their abundance, no corresponding activation of CAF markers in hASCs was observed. This apparent dissociation between increased cytokine exposure and the absence of CAF-like transdifferentiation suggests that elevated inflammatory signaling alone may be insufficient to drive stable CAF commitment. Rather, CAF conversion likely depends on the presence, combination, and temporal dynamics of specific tumor-derived factors, which appear to be lacking or functionally altered in HHP-treated conditioned media. In this context, sustained profibrotic signaling pathways, including but not limited to TGF-β–associated mechanisms, may play a contributory role but were not dominantly activated under the applied experimental conditions. Thus, the observed cytokine secretion is more consistent with a transient inflammatory activation than with durable pro-fibrotic stromal reprogramming.

Interestingly, Al-toub et al. reported that stimulation of MSCs with IL-1β (50 ng/mL), either alone or in combination with IL-6 (50 ng/mL each), strongly upregulated pro-inflammatory cytokines such as IL-1β, CXCL5, IL-6, and CXCL6 at the transcriptional level, whereas IL-6 alone induced only a weak response [63]. In our study, IL-1β concentrations in the conditioned media ranged from 20 to 115 pg/mL in 0 MPa-CM and from 480 to 1220 pg/mL in 300 MPa-CM, corresponding to levels approximately 40- to 2500-fold lower than those used in the Al-toub et al. study. This substantial difference in cytokine concentration likely accounts for the absence of a robust pro-inflammatory or CAF-like conversion in hASCs in our model. A study employing a comparable experimental setup in breast cancer demonstrated that hASCs incubated with TCM from two different breast cancer cell lines for 96 h secreted significantly higher amounts of stromal derived factor 1 (SDF-1) and TGF-β1 than unstimulated cells [50]. This paracrine feedback was accompanied by a more than ten-fold increase in α-SMA–positive cells compared to untreated controls, highlighting a strong reciprocal activation between tumor-derived and stromal cytokines [50]. In contrast, our semi-quantitative cytokine screening did not reveal enhanced secretion of either SDF-1 or TGF-β1 after CM exposure. The corresponding array spots were barely visible in both control and CM-treated samples, suggesting that hASCs did not produce relevant amounts of these cytokines under the respective types of stimulation.

From a translational perspective, these findings are highly relevant for a potential clinical application of HHP-treated tissues in reconstructive surgery following tumor resection. The absence of any indications of hASC-to-CAF transdifferentiation in response to HHP-treated tumor CM suggests that devitalized grafts are unlikely to contribute to a tumor-promoting microenvironment upon re-implantation, even in the presence of residual cytokines within the devitalized cartilage. This directly addresses a key oncological safety concern in the use of HHP for the preparation of autologous cartilage grafts, as residual tumor-derived factors must not stimulate stromal cells in a pro-oncogenic manner. Together with previous evidence demonstrating that HHP treatment preserves tissue integrity and antigenicity while ensuring complete devitalization, and that a tumor-promoting influence on other viable tumor cells can likewise be excluded [11,12,13], our data further strengthen the rationale for its clinically safe implementation. Nevertheless, several limitations of our present in vitro study must be acknowledged. The experiments were performed in a two-dimensional monoculture system that does not fully recapitulate the complexity of the tumor microenvironment. Mechanical cues, ECM stiffness, and the presence of immune cells critically modulate fibroblast activation and CAF plasticity. Furthermore, the incubation period of 96 h may not capture delayed transcriptional or epigenetic reprogramming events. In addition, although the conditioned media were filtered through a 0.2 µm membrane, expected to remove apoptotic bodies and larger vesicular structures, smaller extracellular vesicles (EVs) may persist and contribute to the observed biological effects. Importantly, EV-mediated signaling can modulate cell behavior without necessarily inducing stable CAF-like differentiation; however, the specific contribution of EVs following HHP treatment was not addressed in the present study and warrants further investigation. Future studies should incorporate three-dimensional collagen or hydrogel matrices, longer stimulation periods, and co-cultures with immune cells to better approximate in vivo conditions. In addition, the use of primary HNSCC cells instead of established cell lines would provide a more physiologically relevant cytokine milieu and better reflect the intertumoral heterogeneity observed in patient tumors. The combination of such approaches with HNSCC organoid models or animal implantation studies could further validate the oncological safety of HHP treatment under more representative conditions.

In conclusion, this study demonstrates that while hASCs retain the capacity to adopt a CAF-like phenotype under strong stimuli such as TGF-β1, HHP-treated tumor supernatants fail to induce such changes under the applied in vitro conditions. HHP devitalization substantially reduces the paracrine tumor-promoting potential of malignant cells while preserving extracellular matrix integrity. Collectively, these findings provide preclinical in vitro evidence supporting the oncological safety of HHP-treated tissues. Accordingly, HHP treatment may represent a promising tissue-preserving strategy; however, further in vitro and in vivo investigations are warranted to confirm these observations under physiologically relevant conditions and to assess the translational potential of HHP-treated tissues in clinical oncology.

## Data Availability

The original contributions presented in this study are included in the article/Supplementary Material. Further inquiries can be directed to the corresponding authors.

## Supplementary Materials

The following supporting information can be downloaded at: https://www.mdpi.com/article/10.3390/cimb48010091/s1.

## Abbreviations

The following abbreviations are used in this manuscript:

HHP	High hydrostatic pressure
HNSCC	head and neck squamous cell carcinoma
IL	Interleukin
TME	tumor microenvironment
CAF	cancer-associated fibroblast
EMT	epithelial-to-mesenchymal transition
MSC	mesenchymal stromal cells
hASC	adipose stromal cells
TGF-β1	transforming growth factor beta 1
TNF-α	tumor necrosis factor alpha
CXCR4	C-X-C chemokine receptor 4
CXCL5/12	C-X-C motif chemokine ligand 5/12
STAT3	signal transducer and activator of transcription 3
NF-κB	nuclear factor kappa B
αSMA	alpha-smooth muscle actin
FAP	fibroblast activation protein
TNC	tenascin-C
SPARC	secreted protein acidic and rich in cysteine
VIM	vimentin
TIMP1	tissue inhibitor of metalloproteinases 1
DMEM	Dulbecco’s modified Eagel medium
PDX	patient-derived xenograft
HPV	Human papillomavirus
P/S	Penicillin/Streptomycin
EDTA	ethylenediaminetetraacetic acid
ITS	Insulin-Transferrin-Selenium
CTRL	control
CM	Conditioned medium
HHP-CM	High hydrostatic pressure–treated conditioned medium
TCM	tumor-conditioned medium
qRT-PCR	quantitative real-time PCR
cDNA	cComplementary DNA
GAPDH	Glyceraldehyde-3-phosphate dehydrogenase
PFA	paraformaldehyde
BSA	bovine serum albumin
RT	room temperature
MFI	mean fluorescence intensity
SD	standard deviation
ELISA	Enzyme-linked immunosorbent assay
4-PL	Four-parameter logistic
MCP-1	Monocyte Chemoattractant Protein-1
CCL5	CC-chemokine ligand 5
SDF-1	stromal derived factor 1
EV	extracellular vesicle
