# Supplementary material for: Oncological Safety of High Hydrostatic Pressure Treatment: Effects on Cancer-Associated Fibroblast-like Transdifferentiation of Adipose Stromal Cells
Source: Curr Issues Mol Biol. 2026 Jan 16;48(1):91. doi: 10.3390/cimb48010091 (PMC12839575; doi:10.3390/cimb48010091)
Supplement: Supplementary file 1 [file cimb-48-00091-s001.zip › cimb-4073455-supplementary.pdf]

current issues in molecular biology

MDPI

Supplement

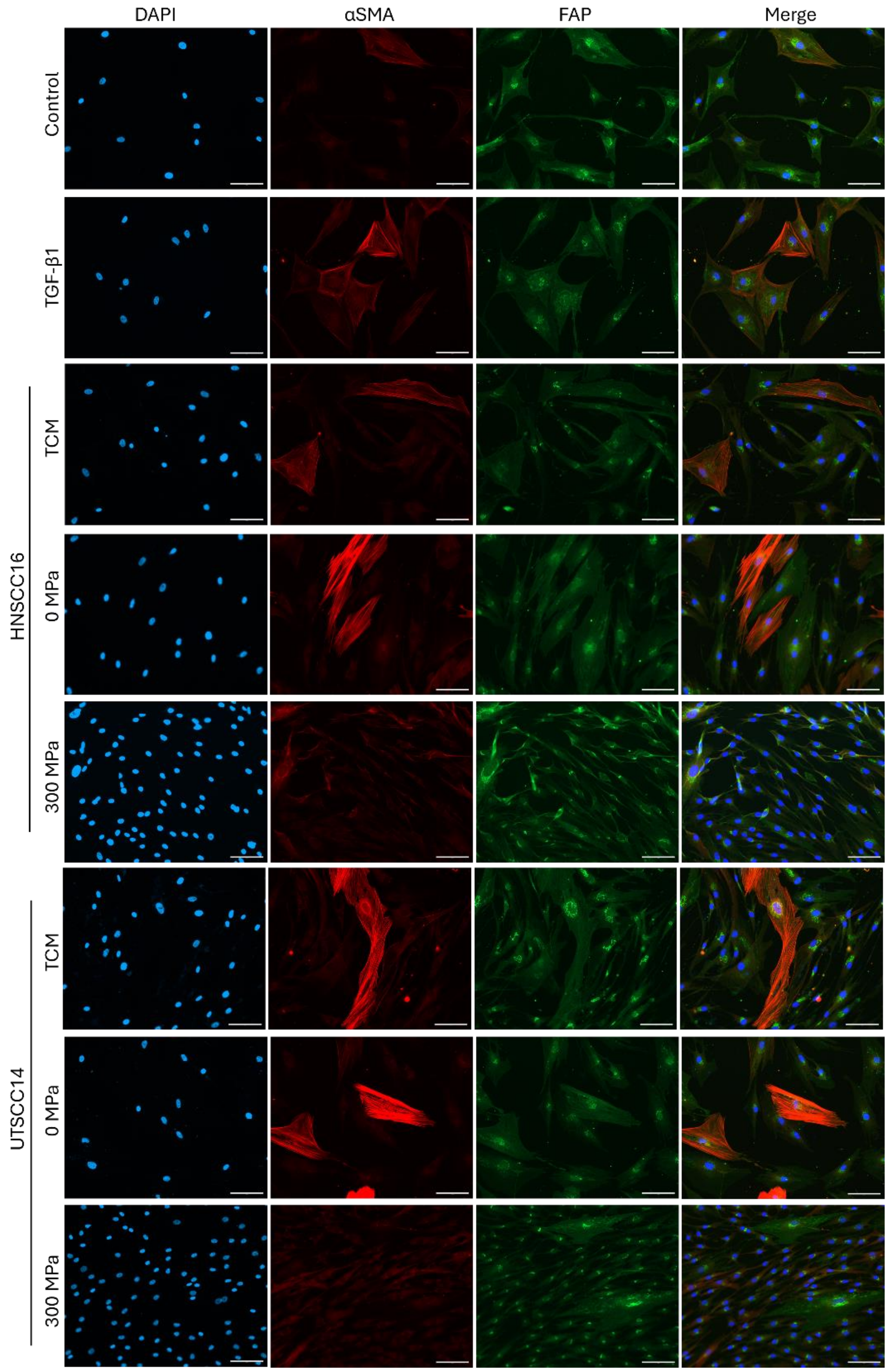

Supplementary Figure S1. Immunofluorescence analysis of α-SMA and FAP in stimulated human adipose stromal cells (hASCs). hASCs were incubated for 96 h with tumor-conditioned medium (TCM), 0 MPa–treated medium, or 300 MPa–treated medium derived from HNSCC16 or UTSCC14 cells, TGF-β1 (10 ng/mL) or serum-free control medium respectively. Representative immunofluorescence images showing staining of cancer-associated fibroblast (CAF) markers α-SMA (red, intracellular) and FAP (green, membrane-associated) with nuclear counterstaining us-ing Hoechst (blue). Scale bar: 100 μm.

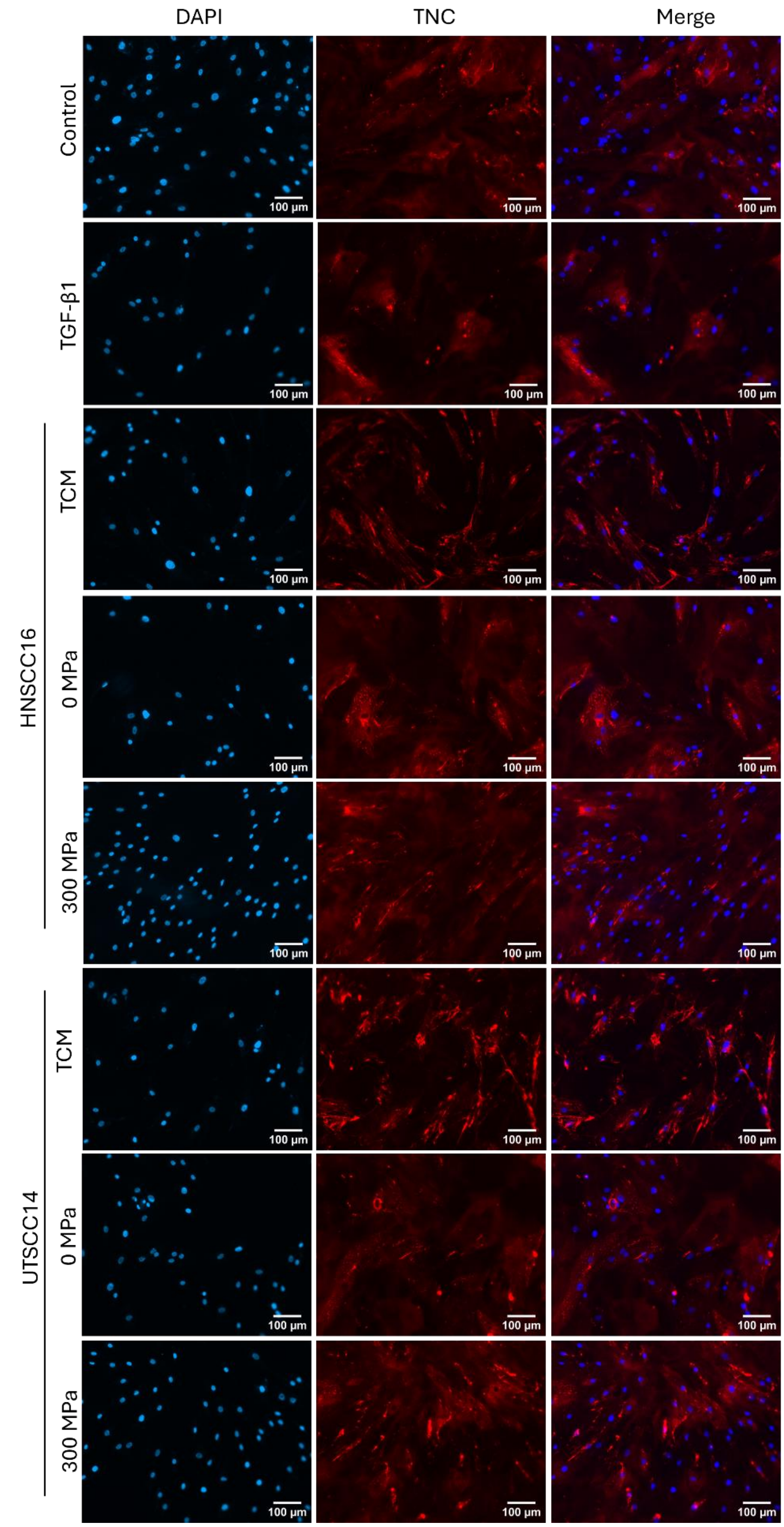

Supplementary Figure S2. Immunofluorescence analysis of TNC in stimulated human adipose stro-mal (hASCs). hASCs were incubated for 96 h with tumor-conditioned medium (TCM), 0 MPa–treated medium, or 300 MPa–treated medium derived from HNSCC16 or UTSCC14 cells, TGF-β1 (10 ng/mL) or serum-free control medium respectively. Representative immunofluorescence stain-ing of TNC (red) and Hoechst (blue) after 96 h of stimulation. Scale bar: 100 µm.

https://doi.org/10.3390/xxxxx

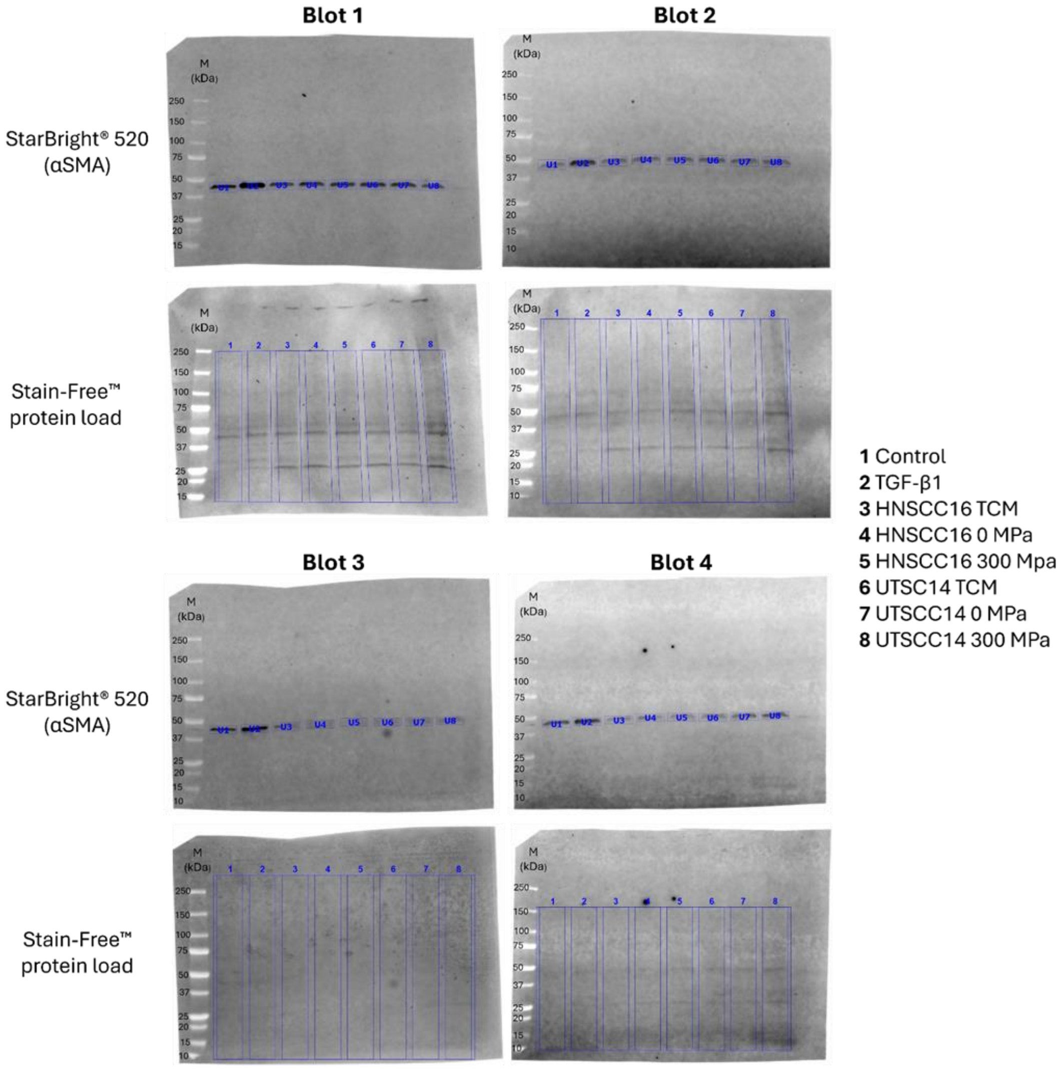

Supplementary Figure S3. Protein quantification of α-SMA by Western blot analysis. α-SMA pro-tein expression was determined by Western blot analysis. Western blot membranes of four donors were analyzed. Precision Plus Protein™ All Blue prestained standards served as molecular weight markers (M). Densitometric quantification of α-SMA protein expression was performed and total protein normalization was performed using Bio-Rad Stain-Free™ technology.

https://doi.org/10.3390/xxxxx

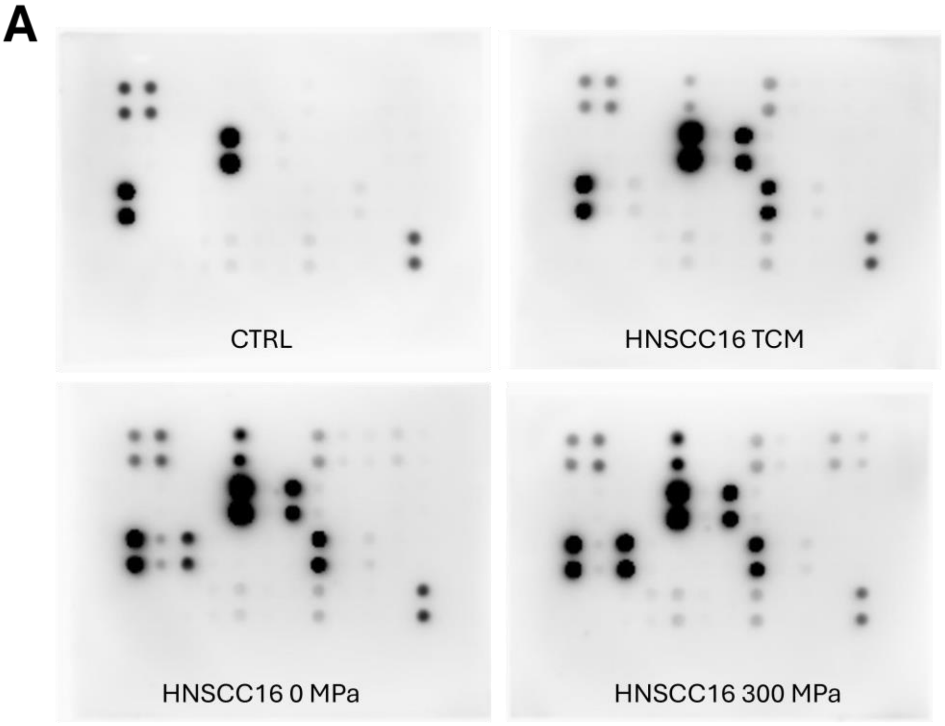

B

POS	POS	NEG	NEG	CXCL5	G-CSF	GM-CSF	GRO	CXCL1	I-309	IL-1α	IL-1β
POS	POS	NEG	NEG	CXCL5	G-CSF	GM-CSF	GRO	CXCL1	I-309	IL-1α	IL-1β
IL-2	IL-3	IL-4	IL-5	IL-6	IL-7	IL-8	IL-10	IL-12 p40/70	IL-13	IL-15	INF-γ
IL-2	IL-3	IL-4	IL-5	IL-6	IL-7	IL-8	IL-10	IL-12 p40/70	IL-13	IL-15	INF-γ
MCP-1	MCP-2	MCP-3	M-CSF	CCL22	MIG	MIP-1 delta	CCL5	SCF	SDF-1	TARC	TGF-β1
MCP-1	MCP-2	MCP-3	M-CSF	CCL22	MIG	MIP-1 delta	CCL5	SCF	SDF-1	TARC	TGF-β1
TNFα	TNFβ	EGF	IGF-1	ANG	OSM	TPO	VEGF-A	PDGF-BB	Leptin	NEG	POS
TNFα	TNFβ	EGF	IGF-1	ANG	OSM	TPO	VEGF-A	PDGF-BB	Leptin	NEG	POS

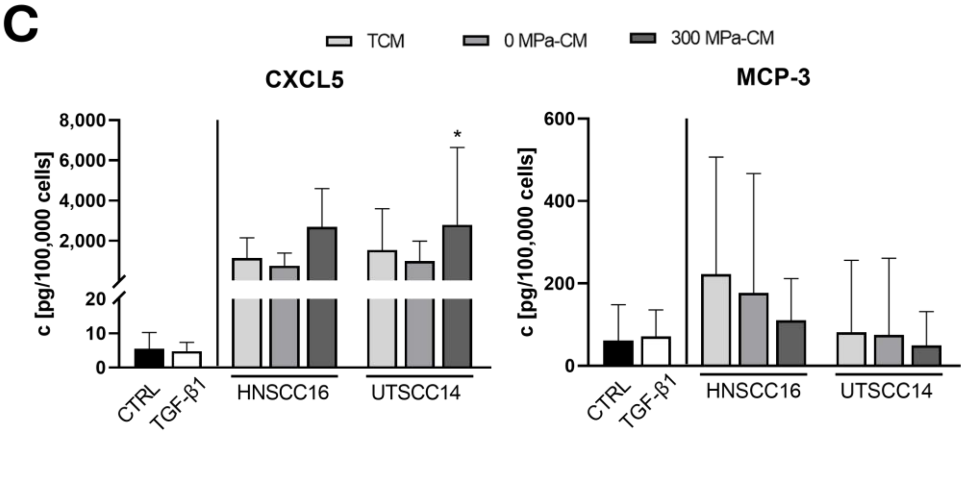

Supplementary Figure S4. Identification of cytokines secreted by human adipose stromal (hASCs) after incubation with conditioned media. hASCs were incubated for 96 h with tumor-conditioned medium (TCM), 0 MPa–treated medium, or 300 MPa–treated medium derived from HNSCC16 or

UTSCC14 cells, TGF-β1 (10 ng/mL), or control medium respectively. After 48 h, the medium was replaced with fresh CM, and the supernatants from the second 48 h incubation period were collected for cytokine analysis. **A** Cytokine array (Dot blot) showing cytokine profiles of serum-free control medium, tumor-conditioned medium (TCM, HNSCC16), 0 MPa–treated CM (HNSCC16), and 300 MPa–treated CM (HNSCC16) after incubation with hASCs. **B** Reference map identifying cytokine positions on the array. **C** Quantification of selected cytokines (MCP-1, IL-6, IL-8, CCL5) in hASC supernatants was determined by multiplex bead-based assay. Values represent the mean ± SD of n = 8 independent donors. Cytokine concentrations measured before and after incubation with hASCs were compared, and the difference (post–pre) normalized to 100,000 cells is shown. Statistical significance was assessed using predefined post hoc comparisons following one-way ANOVA or Kruskal–Wallis testing. Differences were evaluated relative to the control group or between specific treatment conditions as indicated in the figures. Statistically significant differences are indicated by asterisks (*$p < 0.05$).
